# A comparison of the Airtraq^®^, McGrath^®^, and Macintosh laryngoscopes for difficult paediatric intubation: A manikin study

**DOI:** 10.1371/journal.pone.0171889

**Published:** 2017-02-10

**Authors:** Gen Owada, Takahiro Mihara, Gaku Inagawa, Ayako Asakura, Takahisa Goto, Koui Ka

**Affiliations:** 1 Department of Anaesthesiology, Kanagawa Children’s Medical Centre, Yokohama, Japan; 2 Department of Anaesthesiology and Critical Care Medicine, Yokohama City University Graduate School of Medicine, Yokohama, Japan; 3 Department of Anaesthesiology, Yokohama Municipal Citizen’s Hospital, Yokohama, Japan; National Yang-Ming University, TAIWAN

## Abstract

**Background:**

The efficacy of devices for difficult intubation in paediatric patients, especially with a Cormack-Lehane grade 4 view, has yet to be established. We compared intubating parameters among three devices (the Airtraq^®^, McGrath^®^, and Macintosh laryngoscopes).

**Methods:**

This study is a randomised cross-over trial. Participants were 20 anaesthetists. Each device was tested three times using a paediatric manikin with a Cormack-Lehane grade 4 view. The order to use each device was randomised by a computer-generated random sequence. The primary endpoint was the rate of successful intubation. Secondary endpoints included the time taken to intubate, percentage of glottic opening score, and severity of potential dental trauma.

**Results:**

The successful intubation rates of the Airtraq^®^, McGrath^®^, and Macintosh laryngoscopes were 100%, 72%, and 45%, respectively. The risk ratio of the success rates of Airtraq^®^ compared with McGrath^®^ and Macintosh laryngoscopes were 1.40 (95% CI; 1.19–1.64, P < 0.001) and 2.22 (95% CI; 1.68–2.94, P < 0.001), respectively. The modified Cormack-Lehane grade and percentage of the glottic opening score were better for the Airtraq^®^ than for the other devices. The dental trauma score was lower for the Airtraq^®^ than for the other devices. There were no significant differences in the intubation time among the groups.

**Conclusions:**

The Airtraq^®^ had higher success rate, had better visibility, and was associated with less dental trauma than the other devices in a difficult paediatric intubation model with a Cormack-Lehane grade 4 view.

## Introduction

In paediatric patients, failure or taking too much time to intubate in a difficult airway can easily lead to oxygen desaturation due to the patient’s low functional residual capacity, which may cause further complications such as bradycardia or even worse, cardiac arrest [1]. Isada et al. reported that the frequency of difficult intubation is 0.03% in paediatric patients without a specific syndrome, and it increases to 0.18% with congenital syndromes or face anomalies [2]. Recently, devices for difficult intubation (e.g. the Airtraq^®^ or McGrath^®^ MAC video laryngoscope) became available for paediatric patients. However, the efficacy of these devices for difficult intubation in paediatric patients, especially with a Cormack-Lehane (C-L) grade 4 view, has yet to be established, although it has been established for adult patients [3–10]. To our knowledge, only one study by Komiya et al. has evaluated the efficacy of a fibreoptic-assisted laryngoscope in a manikin study with a difficult paediatric intubation model with a C-L grade 4 view [11].

We previously reported the case of a paediatric Emanuel syndrome patient [12] with micrognathia, who was difficult to intubate with the McGrath^®^ laryngoscope (modified C-L [13] [MC-L] grade 4), but was successfully intubated with the Airtraq^®^ device (MC-L grade 1). The Airtraq^®^ does not require a large mandibular space to align the direction of its line of vision with the laryngeal axis because of its steeply curved blade, whereas the McGrath^®^ requires a certain area of mandibular space. Thus, we thought that the Airtraq^®^ is superior to the McGrath^®^ for intubating paediatric patients with severe mandibular hypoplasia.

We hypothesised that Airtraq^®^ would have a higher success rate than the McGrath^®^ and the Macintosh laryngoscopes in difficult paediatric intubation cases. To prove this hypothesis, we compared the efficacy and usability of the Airtraq^®^, McGrath^®^, and Macintosh laryngoscopes using a manikin of a difficult paediatric intubation model with a C-L grade 4 view.

## Materials and methods

This study was reviewed by the institutional ethical committee of the Kanagawa Children’s Medical Centre, Yokohama, Japan. The ethics committee deemed formal approval unnecessary for this study. The study protocol was registered in the UMIN clinical trial registry (registry number: UMIN000014364). Written informed consent was obtained from all study participants. Participants were 20 anaesthetists with at least over 1 year of clinical experience. Their experiences with paediatric anaesthesia were not required.

### The difficult paediatric intubation model and the devices used

**Fig 1:**
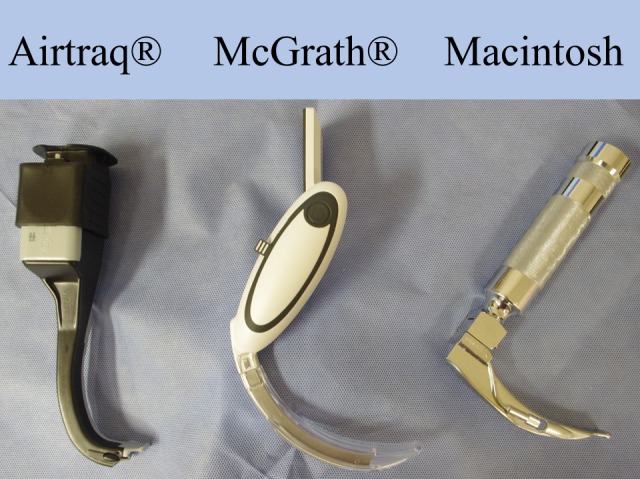
A photo of the Airtraq^®^, McGrath^®^, and Macintosh laryngoscopes used in the study.

The difficult paediatric intubation model (i.e., the C-L grade 4 view manikin) was established in our previous study [11]. The sublingual space (tongue bottom) of a paediatric simulator manikin (MegaCode Kid, Laerdal, Norway) was partially filled with approximately 10 ml of a semi horseshoe-shaped dental impression material (Algiace Z, Dentsply-Sankin, Tokyo, Japan) to reproduce difficult tracheal intubation (i.e., the C-L grade 4 view using a size 2 Macintosh blade). All intubations were performed using an uncuffed tracheal tube (internal diameter, 4.5 mm; Portex, Smiths Medical, Hythe, UK) with an intubating stylet. The size 2 Macintosh blade, size 2 McGrath^®^ blade, and Airtraq^®^ for infant nasal intubation were tested (Fig 1).

### Study protocol

Prior to this study, all participants practiced tracheal intubation using the paediatric manikin of normal airway for three consecutive times, with both the Airtraq^®^ and McGrath^®^ laryngoscopes. The number of intubations was determined based on a previous manikin study[14] investigating the learning curve of the video laryngoscopes. We omitted this process for the Macintosh, because participants were accustomed to using it.

This study is a randomised cross-over trial. Each airway device was used three times (i.e. nine times in total for one anaesthetist). The order to use each device was randomised by a computer-generated random sequence, which was concealed to the participants. Before each intubation trial, the investigator (G.O.) confirmed the C-L grade 4 view of the modified manikin by using a size 2 Macintosh blade, and the investigator fixed the position of the dental impression material as necessary to ensure the C-L grade 4 view. The head position of the manikin were adjusted by the participants.

The primary endpoint was the rate of successful intubation. A successful intubation was defined when the lungs of the manikin were inflated after the tracheal tube was connected to a self-inflating bag. Failed intubation was defined as an intubation time >120 sec or when the operator gave up. Within 120 sec, the operator could try repeatedly until they successfully completed intubation (even after oesophageal intubation). One attempt was defined as the withdrawal of the device from the mouth followed by repositioning. Secondary endpoints included the time for successful intubation, which was defined as the time taken from inserting the blade between the teeth until chest inflation was confirmed; the C-L grade for Macintosh and the MC-L grade (13) for both Airtraq^®^ and McGrath^®^ laryngoscopes; percentage of glottic opening score (POGO score) [15]; number of oesophageal intubations; and severity of potential dental trauma, which was visually graded by the pressure exerted on the upper teeth (0 = none, 1 = moderate: the blade of the device touched the upper teeth, and 2 = severe: the blade of the device bent the upper teeth). At the end of the study, the participants were asked by the investigator which device they would like to use in a similar clinical intubation situation.

### Statistical analysis

From our pilot study, we assumed the following success rates of the Macintosh, McGrath^®^, and Airtraq^®^ laryngoscopes: 0%, 50%, and 100%, respectively. A sample size of 19 in each group would have 80% power to detect a 50% difference in the success rate using Fisher’s exact test with Bonferroni correction, assuming a 0.017 (0.05/3) level of significance. For the statistical test for repeated measures multi-arm data, the success rate and the number of oesophageal intubations were analysed using Cochrane Q test with Bonferroni correction. The time for successful intubation, MC-L grade, POGO scale, and severity of dental trauma were analysed using the Friedman test followed by the Wilcoxon signed rank sum test with Bonferroni correction. The risk ratio with 95% confidence intervals (CI) of the success rate of Airtraq^®^ compared with McGrath^®^ and Macintosh were calculated. Continuous variables were analysed for normal distribution with Shapiro-Wilk’s test. Continuous variables with normal distribution are presented as mean (standard deviation), and those without normal distribution were presented as median (interquartile range); categorical variables are presented as numbers and frequencies with 95% CI. Statistical analyses were performed using the R statistical software package, version 3.0.2 (R Foundation for Statistical Computing, Vienna, Austria). A P value <0.05 was considered significant.

## Results

Of the 20 participants in the current study, seven (35%) were experienced paediatric anaesthetists. Of the other 13 participants, four were anaesthetists with clinical experience of less than five years, and the other nine were anaesthetists with clinical experience of more than five years.

### Primary endpoint

The success rates for each device are shown in Table 1. The success rates (95% CI) of the Airtraq^®^, McGrath^®^, and Macintosh laryngoscopes were 100% (95.1% to 100%), 72% (58.6% to 82.5%), and 45% (32.1% to 58.4%), respectively. The risk ratio of the success rates of Airtraq^®^ compared with McGrath^®^ and Macintosh laryngoscopes were 1.40 (95% CI; 1.19–1.64, P < 0.001) and 2.22 (95% CI; 1.68–2.94, P < 0.001), respectively.

**Table 1:** Results of the three laryngoscopes.

	Airtraq^®^ (n = 20)	McGrath^®^ (n = 20)	Macintosh (n = 20)	P value
Success rate [95% CI] (%)	100 [95.1 to 100]	72 [58.6 to 82.5]	45 [32.1 to 58.4]	<0.001
Time for successful intubation (sec)	40 (30–62)	35 (27–61)^a^	47 (41–57)^b^	0.88
MC-L grade	1 (1–1)	2a (2a–2b)	4 (4–4)	<0.001
POGO score (%)	100 (100–100)	65 (30–80)	0 (0–0)	<0.001
Severity of dental trauma	0 (0–1)	2 (1–2)	2 (2–2)	<0.001

MC-L, modified Cormack-Lehane; POGO, percentage of glottic opening score. a, b: two and six participants were excluded from the data because of intubation failure in all three attempts. Values are presented as median (interquartile range), or percentage [95% confidence interval].

### Secondary endpoints

The intubation time, MC-L grade, POGO score, and severity of potential dental trauma for each device are shown in Table 1. There were no significant differences in the time for successful intubation among the groups (P = 0.88). The MC-L grade was less for the Airtraq^®^ than for the Macintosh (P < 0.001) or McGrath^®^ (P < 0.001). The MC-L grade was less for the McGrath^®^ than for the Macintosh (P < 0.001). The POGO score was better for the Airtraq^®^ than for the Macintosh (P < 0.001) or the McGrath^®^. The POGO score was better for the McGrath^®^ than for the Macintosh (P < 0.001). The dental trauma score was lower for the Airtraq^®^ than for the Macintosh (P < 0.001) or the McGrath^®^ (P < 0.001). There were no significant differences in the dental trauma score between the Macintosh and McGrath^®^ (P = 0.21).

The numbers of oesophageal intubations performed using the Airtraq^®^, McGrath^®^, and Macintosh were 1 (1.6%), 0 (0%), and 12 (20%), respectively. There were no significant differences in the number of oesophageal intubations performed between the Airtraq^®^ and the McGrath^®^ (P = 1.0). More oesophageal intubations were performed with the Macintosh than with the Airtraq^®^ (P < 0.001) or the McGrath^®^ (P < 0.001).

Among 20 anaesthetists who completed this study, 13 preferred to use the Airtraq^®^ (65%), 7 preferred to use the McGrath^®^ (35%), and none chose to use the Macintosh in a similar clinical intubation situation. There were no significant differences in the preference of devices between the Airtraq^®^ and the McGrath^®^ (P = 0.34). The Airtraq^®^ was preferred over the Macintosh (P < 0.001). Also, the McGrath^®^ was preferred over the Macintosh (P < 0.025).

All data of the current study are provided in S1 Table.

## Discussion

In the current manikin study with a difficult paediatric intubation model with a C-L grade 4 view, we demonstrated that the Airtraq^®^ had a higher success rate than the McGrath^®^ or Macintosh laryngoscopes. Additionally, the Airtraq^®^ revealed better visibility and less dental trauma than the other devices. However, there were no differences among these devices regarding the time for successful intubation.

The Airtraq^®^ is advantageous in terms of the success rate of intubation, MC-L grade, and POGO score, probably due to its steeper curved blade than the other devices. The modified manikin with a C-L grade 4 view had a narrow oral space and retracted tongue root for dental impression material. As a result, gently curved blades of the McGrath^®^ and Macintosh laryngoscopes (Fig 1) could not align the direction of its line of vision with the laryngeal axis. In contrast, the steep curved blade of the Airtraq^®^ could align the direction of its line of vision with the laryngeal axis. Previous study [16] reported that the POGO score was better, but first attempt intubation success rate was lower with the Airtraq^®^ than Macintosh laryngoscopy in paediatric patients with normal airway anatomy. Although our manikin study suggested that the Airtraq^®^ may provide higher success rate than McGrath^®^ or Macintosh laryngoscopy, clinical studies in paediatric patients with difficult airway are needed.

A lower dental trauma score was observed for Airtraq^®^. This can also be explained by the shape of the blade and the difference in the way that the larynx is visualized among these devices. Although the Airtraq^®^ can visualize the larynx without requiring force, the McGrath^®^ and Macintosh laryngoscopes need to lever back excessively to align the direction of its line of vision with the laryngeal axis in the modified manikin, which leads to the dental trauma. In a previous study that used a manikin for a difficult adult intubation model, there was no significant difference between the Airtraq^®^ and McGrath^®^ laryngoscopes in terms of dental trauma [9]. However, in more difficult intubation cases with an MC-L grade 4, there was a significant difference in terms of dental trauma.

There were no significant differences in the time for successful intubation among all the devices. This may be due to the narrow intraoral and pharyngeal space of the manikin. As previously stated, the Airtraq^®^ had better visibility. However, there was no adequate space to insert and control the tracheal tube. Consequently, participants had to take much time to intubate, even though they could visualize the larynx. The time for successful intubation was calculated using only the successful intubation cases (i.e., 100%, 72%, and 45% of data in the Airtraq^®^, McGrath^®^, and Macintosh groups, respectively). Therefore, the result regarding intubation time probably does not represent the true intubation time in real world. Although the time for intubation including failure cases may be shorter for the Airtraq^®^ compared to McGrath^®^ or Macintosh, we could not make any conclusion about it.

In the present study, an intubating stylet was used in all the trials. Firstly because, it is common to use the intubating stylet for tracheal intubation by the Macintosh or the McGrath^®^ in difficult intubation situations. Secondly, we referred to the report by Xue et al. which used the nasotracheal Airtraq^®^ with the intubating stylet for difficult orotracheal intubation [17]. Our findings could not be directly applicable to the Airtraq^®^ for oral intubation which has guide rail and do not need the intubating stylet.

Our study has some limitations. Firstly, this study was performed on a manikin, not a human patient. As there have been some reports about the differences between manikins and human patients [18], the manikin in this study may not precisely reproduce a difficult airway in a paediatric patient. However, it is common to assess new airway devices in a manikin first with ethical concerns of using them in patients [3,4]. Furthermore, the airway with an MC-L grade 4 view is so rare that systematic evaluation of the airway device is difficult, and it poses many health risks. Secondly, this study was unblinded. Blinding was unrealistic because it was very difficult to hide the airway devices from the investigator and the participants who attempted intubation. Thus, the results were not free from observer bias. However, our primary endpoint (i.e. the success rate of the devices) was an objective measure, and thus it would be unlikely to be affected by observer bias. Thirdly, our results may be confounded by the learning curve effect of Airtraq^®^ or McGrath^®^. The success rate may improve after accumulation of experience in using these devices. Fourthly, a majority of the participants in this study were not expert paediatric anaesthetists. Therefore, our results may not be applicable to expert paediatric anaesthetists. Finally, there was a discrepancy between the success rate of our pilot study and that of the current study, especially for the Macintosh laryngoscope. Therefore, our sample size calculation may have been inaccurate. However, the positive results obtained as the difference among the three devices regarding their success rates indicated that type II error was not suspected in this study.

In conclusion, our manikin study showed that the success rate is higher for the Airtraq^®^ than for the McGrath^®^ or Macintosh laryngoscope in a difficult paediatric intubation model with a C-L grade 4 view. The Airtraq^®^ has advantages in terms of the MC-L grade, POGO score, and dental trauma. The use of this device in the clinical setting and in human patients should be addressed in future studies.

## Supporting information

S1 Table Data of the current study. (DOCX)
