# Supplementary material for: A comparison of the Airtraq®, McGrath®, and Macintosh laryngoscopes for difficult paediatric intubation: A manikin study
Source: PLoS One. 2017 Feb 10;12(2):e0171889. doi: 10.1371/journal.pone.0171889 (PMC5302788; doi:10.1371/journal.pone.0171889)
Supplement: S1 Table — (DOCX) [file pone.0171889.s001.docx]

Data were arranged in the following order from left; Participant number, Sequence, Group (devise), Success, time (sec), number of intubation attempt, mC-L, POGO score, device, intubation of esophagus, teeth injury score, preferred device

1 1 Macintosh 1 47 1 4 0 0 2

1 2 Macintosh 0 NA NA 4 0 2 2

1 3 Macintosh 0 NA NA 4 0 2 2

1 1 Airtraq 1 40 1 1 100 0 1

1 2 Airtraq 1 33 1 1 100 0 0

1 3 Airtraq 1 34 1 1 100 0 0

1 1 McGrath 1 64 1 2 80 0 2

1 2 McGrath 0 NA NA 2 40 0 2

1 3 McGrath 0 NA NA 2.5 10 0 2 Airtraq

2 1 Macintosh 0 NA NA 4 0 0 1

2 2 Macintosh 0 NA NA 4 0 1 1

2 3 Macintosh 1 18 1 2.5 60 0 1

2 1 Airtraq 1 40 1 1 100 0 1

2 2 Airtraq 1 29 1 1 100 0 0

2 3 Airtraq 1 59 1 1 100 0 1

2 1 McGrath 0 NA NA 2.5 30 0 1

2 2 McGrath 1 29 1 2 60 0 1

2 3 McGrath 1 43 1 2 80 0 1 Airtraq

3 1 Macintosh 1 29 1 4 0 0 2

3 2 Macintosh 1 56 1 4 0 0 2

3 3 Macintosh 1 18 1 4 0 0 2

3 1 Airtraq 1 57 1 1 100 0 1

3 2 Airtraq 1 24 1 1 100 0 1

3 3 Airtraq 1 23 1 1 100 0 1

3 1 McGrath 1 119 2 2 80 0 2

3 2 McGrath 1 32 1 2 50 0 1

3 3 McGrath 1 35 1 2 40 0 2 Airtraq

4 1 Macintosh 1 95 1 4 40 0 2

4 2 Macintosh 1 54 1 3 20 0 2

4 3 Macintosh 1 41 1 3 20 0 2

4 1 Airtraq 1 62 1 1 100 0 1

4 2 Airtraq 1 32 1 1 100 0 1

4 3 Airtraq 1 35 1 1 100 0 1

4 1 McGrath 0 NA NA 2 75 1 2

4 2 McGrath 1 111 2 2 70 0 2

4 3 McGrath 1 23 1 1 90 0 2 Airtraq

5 1 Macintosh 1 93 2 4 0 0 2

5 2 Macintosh 1 52 1 4 0 0 2

5 3 Macintosh 0 NA NA 4 0 0 2

5 1 Airtraq 1 49 1 1 100 0 1

5 2 Airtraq 1 49 1 1 80 0 1

5 3 Airtraq 1 114 1 1 100 0 1

5 1 McGrath 1 98 1 2.5 30 0 2

5 2 McGrath 0 NA NA 4 0 0 2

5 3 McGrath 0 NA NA 4 0 0 1 Airtraq

6 1 Macintosh 1 55 1 4 0 0 2

6 2 Macintosh 0 NA NA 4 0 0 2

6 3 Macintosh 1 61 1 4 0 0 2

6 1 Airtraq 1 36 1 1 100 0 0

6 2 Airtraq 1 31 1 1 100 0 0

6 3 Airtraq 1 27 1 1 100 0 1

6 1 McGrath 1 58 1 2.5 30 0 2

6 2 McGrath 1 54 1 2 80 0 2

6 3 McGrath 1 28 1 2 80 0 2 Airtraq

7 1 Macintosh 1 44 1 4 0 0 2

7 2 Macintosh 1 59 1 4 0 0 2

7 3 Macintosh 1 43 1 4 0 0 2

7 1 Airtraq 1 55 1 1 100 0 0

7 2 Airtraq 1 48 1 1 100 0 0

7 3 Airtraq 1 30 1 1 100 0 0

7 1 McGrath 1 40 1 2 50 0 2

7 2 McGrath 0 NA NA 4 0 0 2

7 3 McGrath 0 NA NA 4 0 0 2 Airtraq

8 1 Macintosh 0 NA NA 4 0 1 2

8 2 Macintosh 1 41 1 4 0 0 2

8 3 Macintosh 0 NA NA 4 0 2 2

8 1 Airtraq 1 119 2 1 100 0 1

8 2 Airtraq 1 59 1 1 100 0 1

8 3 Airtraq 1 85 1 1 100 0 0

8 1 McGrath 1 66 1 1 100 0 1

8 2 McGrath 1 63 1 2.5 80 0 1

8 3 McGrath 1 82 1 2 70 0 1 Airtraq

9 1 Macintosh 0 NA NA 4 0 0 2

9 2 Macintosh 1 73 1 4 0 0 2

9 3 Macintosh 1 58 1 4 0 0 2

9 1 Airtraq 1 92 1 1 100 0 0

9 2 Airtraq 1 42 1 1 100 0 0

9 3 Airtraq 1 74 1 1 100 0 0

9 1 McGrath 1 32 1 2 80 0 2

9 2 McGrath 1 21 1 1 100 0 2

9 3 McGrath 1 24 1 1 100 0 2 McGrath

10 1 Macintosh 0 NA NA 4 0 0 2

10 2 Macintosh 0 NA NA 4 0 0 2

10 3 Macintosh 0 NA NA 4 0 0 1

10 1 Airtraq 1 97 1 1 100 0 0

10 2 Airtraq 1 62 1 1 100 0 0

10 3 Airtraq 1 61 1 1 100 0 0

10 1 McGrath 0 NA NA 4 0 0 0

10 2 McGrath 0 NA NA 4 0 0 0

10 3 McGrath 0 NA NA 4 0 0 1 Airtraq

11 1 Macintosh 1 50 1 2.5 20 0 2

11 2 Macintosh 1 43 1 2.5 20 0 2

11 3 Macintosh 1 35 1 2.5 30 0 2

11 1 Airtraq 1 26 1 1 100 0 1

11 2 Airtraq 1 20 1 1 100 0 0

11 3 Airtraq 1 18 1 1 100 0 0

11 1 McGrath 1 27 1 1 100 0 2

11 2 McGrath 1 20 1 1 100 0 2

11 3 McGrath 1 22 1 1 100 0 2 McGrath

12 1 Macintosh 0 NA NA 4 0 0 2

12 2 Macintosh 0 NA NA 4 0 0 2

12 3 Macintosh 0 NA NA 4 0 0 2

12 1 Airtraq 1 35 1 1 100 0 0

12 2 Airtraq 1 26 1 1 100 0 0

12 3 Airtraq 1 20 1 1 100 0 0

12 1 McGrath 0 NA NA 2.5 20 0 0

12 2 McGrath 1 67 1 2.5 40 0 0

12 3 McGrath 0 NA NA 2.5 10 0 0 Airtraq

13 1 Macintosh 0 NA NA 4 0 0 0

13 2 Macintosh 0 NA NA 4 0 0 0

13 3 Macintosh 0 NA NA 4 0 0 0

13 1 Airtraq 1 47 1 1 100 0 0

13 2 Airtraq 1 62 1 1 100 0 0

13 3 Airtraq 1 46 1 1 100 0 0

13 1 McGrath 0 NA NA 4 0 0 0

13 2 McGrath 0 NA NA 4 0 0 1

13 3 McGrath 0 NA NA 4 0 0 1 Airtraq

14 1 Macintosh 0 NA NA 4 0 1 1

14 2 Macintosh 0 NA NA 4 0 0 1

14 3 Macintosh 0 NA NA 4 0 0 2

14 1 Airtraq 1 30 1 1 100 0 1

14 2 Airtraq 1 26 1 1 100 0 0

14 3 Airtraq 1 26 1 1 100 0 0

14 1 McGrath 1 72 1 2 40 0 2

14 2 McGrath 1 58 1 2 40 0 2

14 3 McGrath 1 33 1 2 70 0 2 Airtraq

15 1 Macintosh 0 NA NA 4 0 0 2

15 2 Macintosh 1 36 1 4 0 0 2

15 3 Macintosh 1 27 1 4 0 0 2

15 1 Airtraq 1 37 1 1 100 0 0

15 2 Airtraq 1 30 1 1 100 0 1

15 3 Airtraq 1 71 1 1 100 0 1

15 1 McGrath 1 51 1 2.5 40 0 2

15 2 McGrath 1 25 1 2.5 40 0 2

15 3 McGrath 1 17 1 2.5 50 0 2 McGrath

16 1 Macintosh 0 NA NA 4 0 0 0

16 2 Macintosh 0 NA NA 4 0 0 0

16 3 Macintosh 0 NA NA 4 0 0 2

16 1 Airtraq 1 110 1 1 100 0 0

16 2 Airtraq 1 56 1 1 100 0 0

16 3 Airtraq 1 74 1 1 100 0 0

16 1 McGrath 1 39 1 2.5 15 0 1

16 2 McGrath 1 33 1 2 80 0 0

16 3 McGrath 1 25 1 1 100 0 1 McGrath

17 1 Macintosh 0 NA NA 4 0 0 1

17 2 Macintosh 0 NA NA 4 0 0 0

17 3 Macintosh 0 NA NA 4 0 0 0

17 1 Airtraq 1 24 1 2 50 0 0

17 2 Airtraq 1 18 1 2 40 0 0

17 3 Airtraq 1 33 1 2 80 0 0

17 1 McGrath 1 55 1 2 80 0 1

17 2 McGrath 0 NA NA 2 40 0 0

17 3 McGrath 1 23 1 2 70 0 2 Airtraq

18 1 Macintosh 0 NA NA 4 0 0 2

18 2 Macintosh 1 52 1 4 0 0 2

18 3 Macintosh 0 NA NA 4 0 0 2

18 1 Airtraq 1 119 1 1 100 0 0

18 2 Airtraq 1 37 1 1 100 0 0

18 3 Airtraq 1 37 1 1 100 0 0

18 1 McGrath 1 102 1 2.5 40 0 2

18 2 McGrath 1 55 1 2 70 0 2

18 3 McGrath 1 36 1 2 80 0 2 McGrath

19 1 Macintosh 0 NA NA 4 0 2 2

19 2 Macintosh 0 NA NA 4 0 1 2

19 3 Macintosh 1 43 1 4 0 0 2

19 1 Airtraq 1 77 1 1 100 0 1

19 2 Airtraq 1 30 1 1 100 0 0

19 3 Airtraq 1 34 1 1 100 0 1

19 1 McGrath 1 30 1 1 100 0 2

19 2 McGrath 1 28 1 1 100 0 2

19 3 McGrath 1 26 1 1 100 0 2 McGrath

20 1 Macintosh 0 NA NA 4 0 1 2

20 2 Macintosh 1 72 2 4 0 1 2

20 3 Macintosh 1 47 1 3 0 0 2

20 1 Airtraq 1 81 1 1 100 0 1

20 2 Airtraq 1 91 1 1 100 0 0

20 3 Airtraq 1 40 1 1 100 0 0

20 1 McGrath 1 66 1 2 70 0 1

20 2 McGrath 1 24 1 2 80 0 2

20 3 McGrath 1 30 1 2 80 0 1 McGrath
